# *Melicope balgooyi* Appelhans, W.L. Wagner & K.R. Wood, a new species and new record in *Melicope* section *Melicope* (Rutaceae) for the Austral Islands

**DOI:** 10.3897/phytokeys.39.7691

**Published:** 2014-07-25

**Authors:** Marc S. Appelhans, Warren L. Wagner, Kenneth R. Wood

**Affiliations:** 1 Department of Systematic Botany, Albrecht-von-Haller Institute of Plant Sciences, University of Göttingen, Untere Karspüle 2, 37073 Göttingen, Germany; 2 Department of Botany, Smithsonian Institution, PO Box 37012, Washington, DC 20013-7012, USA; 3 National Tropical Botanical Garden, 3530 Papalina Road, Kalaheo, HI 96741, USA

**Keywords:** Austral Islands, Bass Islands, *Melicope*, French Polynesia, Pacific biogeography, Rapa Iti, Rutaceae, IUCN Red List Category

## Abstract

*Melicope balgooyi*, a new species of *Melicope* (Rutaceae) is described. It is known only from the Austral Islands in the South Pacific (French Polynesia). However, it is not closely related to the other two species previously known from the Austral Islands, which are part of *Melicope* section *Vitiflorae*. The new species belongs to *Melicope* section *Melicope* and is most closely related to species from New Zealand, the Kermadec Islands, and the Society Islands. The new species has alternate to sub-opposite leaves, which is a very rare arrangement in *Melicope* and has only been described for two other species of the genus so far.

## Introduction

**Figure 1.**
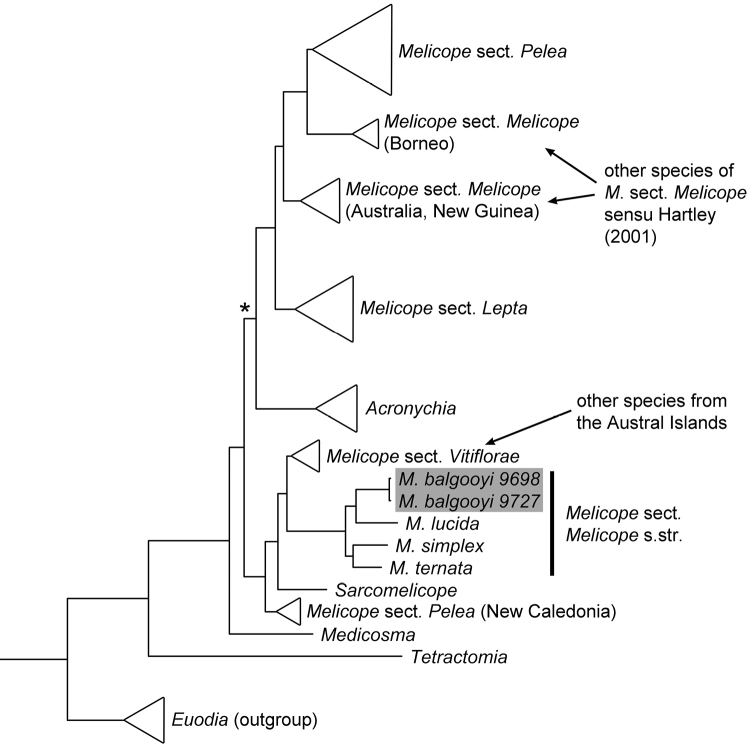
Phylogenetic placement of the newly described *Melicope balgooyi* Appelhans, W.L. Wagner & K.R. Wood is based on an analysis of combined chloroplast and nuclear data (redrawn from Appelhans et al. 2014a). The clade marked with an asterisk (*) was not supported so that the placement of *Acronychia* J.R. Forst. & G. Forst. within or apart from *Melicope* is not certain.

**Figure 2.**
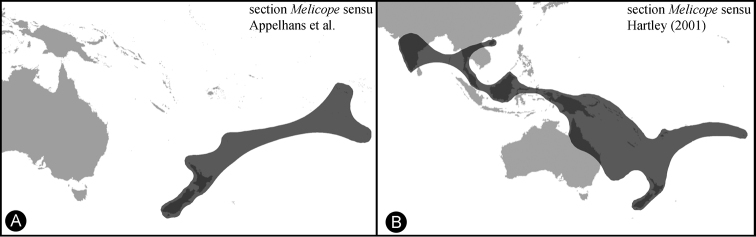
Distribution of *Melicope* section *Melicope*. **A** *Melicope* section *Melicope* sensu Appelhans et al. (2014a), consisting of six species including the newly circumscribed *Melicope balgooyi* from Rapa **B** *Melicope* section *Melicope* sensu Hartley (2001) consisting of 38 species.

In the course of phylogenetic and revisionary studies in *Melicope* J.R. Forst. & G. Forst., a new species was found, and along with its description, we discuss its biogeography and sectional placement. *Melicope* is the largest genus in Rutaceae, consisting of about 235 species divided into the four sections *Lepta* (Lour.) T.G. Hartley, *Melicope*, *Pelea* (A. Gray) Hook. f., and *Vitiflorae* T.G. Hartley (Hartley 2001). Molecular phylogenetic work (Appelhans et al. 2014a) supported Hartley’s (Hartley 1981, Hartley and Stone 1989, Hartley 2001) revisionary work in many ways, however, some of the taxa that were described at the genus level, namely *Comptonella* Baker f., *Dutaillyea* Baill., *Picrella* Baill., *Platydesma* H. Mann, and *Sarcomelicope* Engl., have been shown to belong in *Melicope* (Harbaugh et al. 2009, Appelhans et al. 2014a, 2014b). Also, *Melicope* section *Melicope* has been inferred as non-monophyletic (Appelhans et al. 2014a; Fig. 1). *Melicope* section *Melicope* sensu Hartley (2001) consists of 38 species with a distribution that ranges from India to the Society Islands in the South Pacific (Fig. 2). The section occurs on several Pacific island groups among which are the Bismarck Archipelago, the Salomon Islands, Vanuatu, Fiji, the Society Islands, Lord Howe Island, the Kermadec Islands, and New Zealand. Appelhans et al. (2014a) have found that species in section *Melicope* from Australia, New Guinea, and Borneo do not cluster together with the clade that contains the type species *Melicope ternata* J.R. Forst. & G. Forst. from New Zealand (Fig. 1). Instead, the species from Australia, New Guinea, and Borneo are the closest relatives of *Melicope* section *Pelea* (Appelhans et al. 2014a; Fig. 1). The few species from India, the Malay Peninsula, and Hainan Island (China) were not sampled by Appelhans et al. (2014a), but Hartley (2001) regarded these species as close relatives of the Bornean species. Species from the Bismarck Archipelago, the Salomon Islands, Vanuatu, and Fiji were also not sampled in the study of Appelhans et al. (2014a). Hartley (2001) regarded these species as a closely related group with affinities to species from New Guinea. According to the phylogenetic study by Appelhans et al. (2014a) and the relationships that can be inferred from Hartley’s (2001) revisionary work, the abovementioned taxa have to be excluded from *Melicope* section *Melicope*. A monophyletic section *Melicope* consists only of two species from New Zealand (*Melicope mantellii* Buchanan, *Melicope simplex* A. Cunn.), one species from New Zealand and the Kermadec Islands (*Melicope ternata*), two species from Tahiti (Society Islands; *Melicope lucida* (A. Gray) A.C. Sm., *Melicope tahitiensis* Nadeaud), and the new species described here. All except one of these species were sampled by Appelhans et al. (2014a) and they formed a clade together with two specimens of an undescribed species from Rapa (Rapa Iti, Austral Islands; French Polynesia). *Melicope* section *Melicope* is thus reduced from 38 to six species (Figs 1 and 2).

## Taxonomic treatment

### Melicope balgooyi

Taxon classificationPlantaeSapindalesRutaceae

Appelhans, W.L. Wagner & K.R. Wood sp. nov.

urn:lsid:ipni.org:names:77140886-1

Figs 3 , 4a

#### Type.

AUSTRAL ISLANDS: Rapa Iti, Maii, below rim near Pokumaru, 29 Apr 2002, K.R. Wood 9727 (holotype: PTBG-041326!, isotype: NY!).

This new species of Melicope differs from other species in that genus by the combination of alternate to sub-opposite leaves and oblanceolate leaves with a cordate base.

#### Description.

*Shrub* 50 to 150 cm of height; plants possibly dioecious; trichomes simple, greyish-white; branches brown-red and glabrous, 2–3 mm wide at third internode. *Leaves* with glandular dots, alternate to sub-opposite, unifoliolate, glossy dark green above with yellow or green-white midrib, 4.5–9 × 2.3–3.8 cm, petiole 0–1 mm long, terete, glabrous; blades sub-coriaceous, glabrous on both sides, obovate to oblanceolate, margin entire, apex rounded or obtuse, base cordate; venation brochidodromous, midrib prominulous or plane on both surfaces, secondary veins and veinlet reticulation prominulous, 10–17 secondary veins per side. *Inflorescences* unisexual, axillary, bracteate, several-flowered, up to 1.8 cm long, axes and bracts puberulent. *Flowers* unisexual, tetramerous; pedicel 1.3 to 2.2 mm long; sepals ovate to rounded, pellucid-dotted, glabrous or slightly ciliolate, 1.2 to 1.5 mm long, same size in staminate and pistillate flowers; petals ovate to elliptic, cream-green, pellucid-dotted, glabrous, 2.5 to 2.8 mm long, same size in staminate and pistillate flowers; stamens 8, glabrous; gynoecium 4-carpellate, glabrous, pellucid-dotted, 2 mm long, stigma peltate, young stigma white. *Fruiting* carpels and seeds not known.

**Figure 3.**
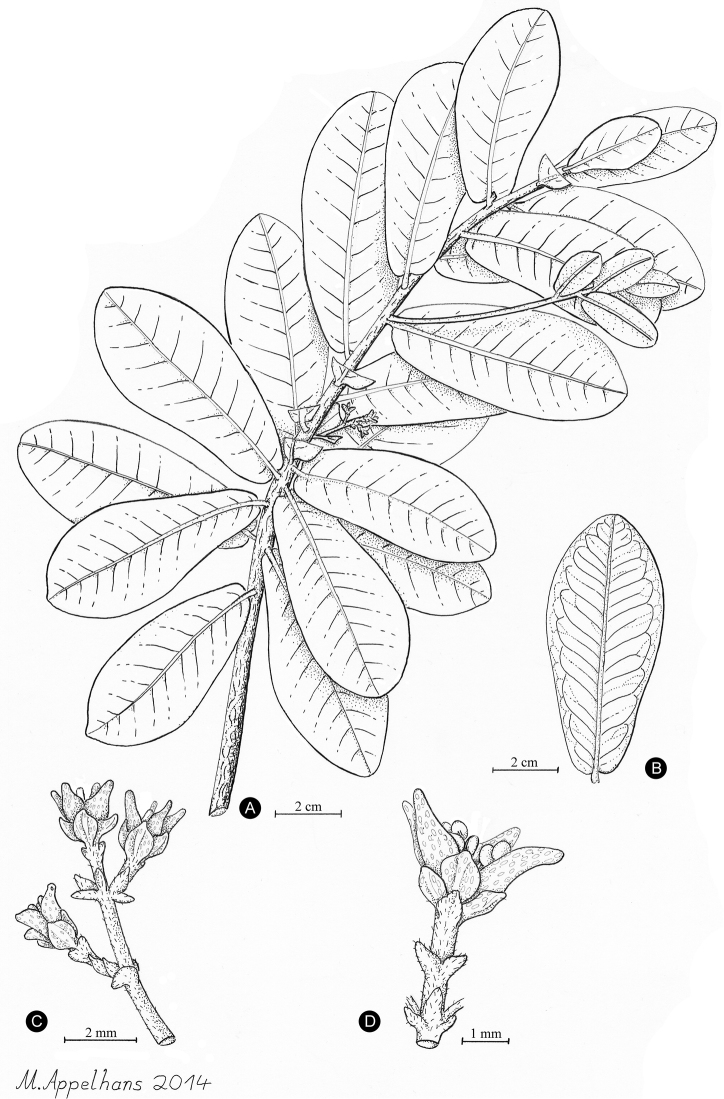
The newly described *Melicope balgooyi* Appelhans, W.L. Wagner & K.R. Wood. **A** Flowering branch **B** Leaf with detailed venation **C** Pistillate inflorescence [peduncle not shown in full length] **D** Single staminate flower. Based on K.R. Wood 9727 (PTBG, **A–C**) and K.R. Wood 9698 (PTBG, **D**).

#### Additional specimens

**(paratypes).** **Austral Islands.** **Rapa:** Pokumaru summit region, upper windswept slopes of Maii, 21 Apr 2002, K.R. Wood 9698 (PTBG!, NY!).

#### Distribution and ecology.

Austral Islands (French Polynesia), only known from the type locality on slopes near the summit of Mount Pokumaru on Rapa; 550-580 m.

**Figure 4.**
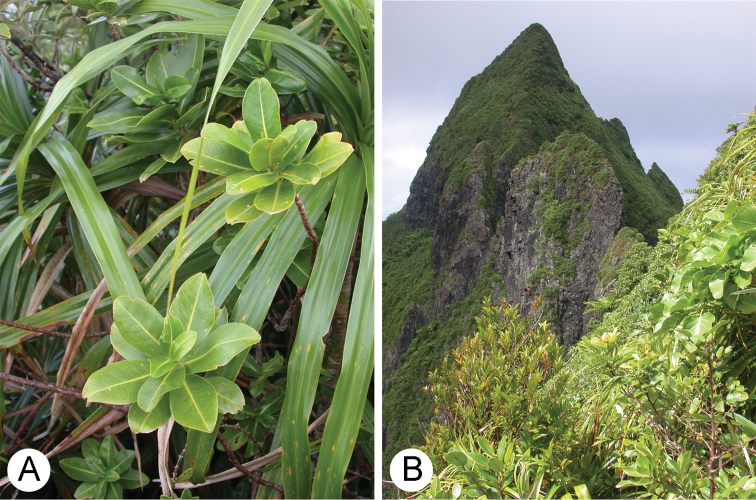
**A** Habit of *Melicope balgooyi* Appelhans, W.L. Wagner & K.R. Wood in situ **B** Habitat of *Melicope balgooyi* around the steep precipitous slopes of Pokumaru, Rapa, Austral Islands.

*Melicope balgooyi* was discovered around the tall spire-like peak of Pokumaru (Fig. 4b) between 550 and 580 m. The habitat is characterized by a windswept shrubland and forest that runs along and below an east to west ridgeline. The plant communities around Pokumaru are unique with small relictual patches of tropical montane cloud forest (TMCF) along with adjacent wet cliffs and steep slopes dominated by *Freycinetia arborea* Gaudich. Tree species in the TMCF zone which are associated with *Melicope balgooyi* include *Fitchia rapense* F. Br., *Meryta choristantha* Harms, *Oparanthus coriaceus* (F. Br.) Sherff, *Carokia collenettei* Riley, *Metrosideros collina* (J.R. Forst. & G. Forst.) A. Gray, *Geniostoma rapense* F. Br., and *Weinmannia rapensis* F. Br. Shrubs, vines, and herbs include *Dianella intermedia* Endl. var. *punctata* F. Br., *Astelia rapensis* Skottsb., *Plantago rupicola* Pilg., *Alyxia stellata* (J.R. Forst. & G. Forst.) Roem. & Schult., *Freycinetia arborea*, and *Hebe rapensis* (F. Br.) Garnock-Jones. Dominant ferns include *Sphaeropteris medullaris* (G. Forst.) Bernh., *Alsophila stokesii* (E.D. Br.) R.M.Tryon, *Blechnum attenuatum* (Sw.) Mett., *Blechnum orientale* L., *Blechnum venosum* Copel., *Blechnum vulcanicum* (Blume) Kuhn var. *rapense* E.D. Br., *Polystichum rapense* E.D. Br., *Dicranopteris linearis* (Burm. F.) Underw., *Belvisia dura* (Copel.) Copel., *Doodia media* R. Br., *Elaphoglossum savaiense* (Baker) Diels, and *Davallia solida* (G. Forst.) Sw. Less than 20 individuals of *Melicope balgooyi* are estimated to occur around this only known site.

#### Etymology.

The species is named in honor of Dr. Max M.J. van Balgooy, a specialist of the SE Asian flora. Dr. van Balgooy has annotated the herbarium sheets of this new species stating that it is an “unusual Rutaceae near *Platydesma*”. Phylogenetic studies (Harbaugh et al. 2009; Appelhans et al. 2014a, 2014b) have shown that *Platydesma* is included within *Melicope*, proving Dr. van Balgooy’s assumptions to be true. In addition to *Melicope maxii* T.G. Hartley, which is endemic to Sulawesi (Indonesia), *Melicope balgooyi* is the second species of *Melicope* named after Dr. van Balgooy.

## Discussion

### Characteristics of the new species and its sectional placement in *Melicope*

The new species from Rapa differs from most *Melicope* species by its alternate to sub-opposite phyllotaxis. While most *Melicope* species are typically opposite-leaved, some species from all sections except *Vitiflorae* can have whorled leaves. This character state is most common on the Hawaiian Islands, where eight of the 52 species (incl. four species of *Platydesma*; Wagner et al. 1990) can have whorled leaves with mainly three to four leaves per node (up to eight leaves per node in *Melicope clusiifolia* (A.Gray) T.G.Hartley & B.C.Stone). Only two *Melicope* species are reported to have sub-opposite or alternate leaves. The New Caledonian *Melicope lasioneura* (Baill.) Baill. ex Guillaumin usually has opposite leaves, but can have leaves in whorls of three or an alternate phyllotaxis. *Melicope rubra* (Lauterb. & K. Schum.) T.G. Hartley from New Guinea and northeastern Australia has opposite or rarely sub-opposite leaves. The alternate to sub-opposite leaves of the new taxon from Rapa therefore represent a rare condition in *Melicope*.

The placement of the new taxon in *Melicope* is assured by molecular phylogenetic data (Appelhans et al. 2014a), which places it close to the type *Melicope ternata* in *Melicope* section *Melicope* (Fig. 1).

Two other species of *Melicope* [*Melicope bracteata* (Nadeaud) S.L. Welsh and *Melicope margaretae* (F. Br.) T.G. Hartley] have been described previously from the Austral Islands, but they are both members of *Melicope* section *Vitiflorae* (Fig. 1). Among other features, the new species differs most distinctly from *Melicope bracteata* and *Melicope margaretae* in stamen number (8) and oblanceolate leaves with a cordate base. *Melicope bracteata* and *Melicope margaretae* each have 4 stamens and usually elliptic leaves with a rounded or acute base.

The new taxon is connected to its closest relatives within *Melicope* section *Melicope* by its 8 stamens. Morphologically, and also phylogenetically, the new taxon mostly resembles the Tahitian *Melicope lucida* and *Melicope tahitiensis*. The often auriculate leaf base of many specimens of *Melicope lucida* and *Melicope tahitiensis* is similar to the cordate leaf base of the new taxon. The leaf shape among the three taxa is also similar; however, *Melicope lucida* and *Melicope tahitiensis* have acuminate to acute apexes as opposed to a rounded or slightly obtuse apex in the new taxon. Leaves of *Melicope lucida* and *Melicope tahitiensis* are petiolate, while those of the new taxon are sessile or subsessile. The new species further shares unisexual flowers and peltate stigmas with *Melicope lucida* and *Melicope tahitiensis*. The holotype specimen has pistillate flowers, while the paratype has staminate flowers, indicating the species may be dioecious like its closest relatives *Melicope lucida* and *Melicope tahitiensis*. However, it is important to note that the description of the new taxon is based on only two collections with a very low number of flowers, so that a definite statement about the sexual system of the species is not possible with the data at hand.

In order to differentiate between *Melicope* and several of its closely related genera on a morphological basis, fruit and seed characters are needed (Hartley 2001, Kubitzki et al. 2011). With fruits lacking on the only known specimens of the new taxon, its position is not absolutely confirmed from a morphological point of view. Consistent with the phylogenetic evidence, further support for the placement of the new taxon in *Melicope* is supported by its distribution. Out of the genera that resemble the flowering and vegetative characters of *Melicope*, most taxa are distributed in Australasia and Malesia (Kubitzki et al. 2011). Only the distribution of *Euodia* J.R. Forst. & G. Forst. reaches deep into the Pacific so that one could expect to find *Euodia* on the Austral Islands. However, the New Caledonian endemic *Euodia tietaensis* (Guillaumin) T.G. Hartley is the only *Euodia* species with eight stamens (like the new species) and all species that occur further eastward in the Pacific have four stamens (Hartley 2001).

### Geology and biogeography

The Austral Islands are part of French Polynesia and are situated in the Southern Pacific. The Archipelago lies south of the Society Islands and consists of seven main islands of volcanic origin. Rapa is the second largest of these islands (i.e. 40 km^2^) and is about 5 million years old. The island is very rugged and is characterized by its steep central ridges, mist shrouded spires, and towering black basalt sea-cliffs. The highest peak is Mont Perau at about 650 m (Gates Clarke 1971, Clouard and Bonneville 2005, Meyer 2010).

The affinities of Rapa’s flora are closely allied to New Zealand and Australia, yet with numerous exceptions. The high levels of endemic biological diversity in both the flora and fauna still puzzle many scientists because of the islands relatively small square area (Wood 2002). Concerning Rapa’s floristic relationships, van Balgooy (1971) stated “I think it best to place Rapa in the SE Polynesian Province, as an anomalous district”. More than 75 plant taxa are single island endemics to Rapa, including three endemic plant genera, namely *Apostates* N. S. Lander (Asteraceae) *Pacifigeron* Nesom (Asteraceae) and *Metatrophis* F. Br. (Urticaceae) (Wood 2002, 2010).

Three species of *Melicope* occur on the Austral Islands, which are the result of two independent colonization events. The newly described species is part of section *Melicope* and its closest relatives stem from Tahiti, the Kermadec Islands, and New Zealand. The two other species, *Melicope bracteata* and *Melicope margaretae*, are part of section Vitiflorae with an origin probably in the area of New Caledonia, Vanuatu, and Fiji based on the distributions of their closest relatives (Hartley 2001, Appelhans et al. 2014a). The new species described here is a new record within section *Melicope* for the Austral Islands and an updated distribution map for the newly revised section is provided in Fig. 2.

## Conservation status

*IUCN Red List Category.* When evaluated using the World Conservation Union (IUCN) criteria for endangerment (IUCN 2001), *Melicope balgooyi* falls into the Critically Endangered (CR) category, which designates this species as facing the highest risk of extinction in the wild. Our evaluation can be summarized by the following IUCN hierarchical alphanumeric numbering system of criteria and subcriteria: B1ab(v); B2a, B2b(i–iii); D. These criteria are defined as: B1, extent of occurrence less than 100 km^2^; B1a, known to exist at only a single location; B1b(v) continuing decline inferred in number of mature individuals; B2, total area of occupancy less than 10 km^2^; B2a, one population known; B2b(i–iii), habitat continuing decline inferred; D, population estimated to number fewer than 50 individuals. Threats to *Melicope balgooyi* include habitat degradation and destruction by feral goats (*Capra hircus* L.), competition with non-native plant taxa especially *Psidium cattleianum* Sabine, possible landslides and fire, and the potential for inbreeding depression from small population.

## Supplementary Material

XML Treatment for Melicope balgooyi
